# The mechanism of action and therapeutic potential of macrophages in osteoporosis: from polarization balance to targeted regulation

**DOI:** 10.3389/fimmu.2026.1738143

**Published:** 2026-05-20

**Authors:** Chuanlong Liu, Tonghao Wang, Zhishuai Ren, Ji Luo, Zhexiang Wang, Yanchen Liang, Bing Peng

**Affiliations:** 1 Department of Orthopedic Trauma Ward I, Hunan Provincial Hospital of Integrated Traditional Chinese and Western Medicine, Changsha, Hunan, China; 2 The First Clinical Medical College, Shandong University of Traditional Chinese Medicine, Jinan, Shandong, China; 3 Department of Orthopedics, The First Affiliated Hospital of Shandong University of Traditional Chinese Medicine, Jinan, Shandong, China; 4 Graduate School, Hunan University of Traditional Chinese Medicine, Changsha, Hunan, China; 5 Department of Orthopedics, Central Hospital, Tianjin University/Tianjin Third Central Hospital, Tianjin, China; 6 Tianjin Key Laboratory of Extracorporeal Life Support for Critical Diseases, Tianjin, China; 7 Tianjin Artificial Cell Engineering Technology Research Center, Tianjin, China; 8 Tianjin Institute of Geriatrics, Tianjin Third Central Hospital Branch, Tianjin, China; 9 Department of Spinal Surgery, Tianjin Union Medical Center, The First Affiliated Hospital of Nankai University, Tianjin, China

**Keywords:** M1-type macrophages, M2-type macrophages, macrophages, osteoimmunology, osteoporosis, polarization balance

## Abstract

Osteoporosis (OP) is a chronic metabolic bone disease characterised by low bone mass and deterioration of bone microarchitecture as its core pathological features, which predominantly affects the elderly population and postmenopausal women. Macrophages, as key components of the immune system, function in regulating inflammatory responses, tissue repair and immune modulation, and play a pivotal role in the pathogenesis and progression of osteoporosis: their distinct polarisation states can directly influence osteoblast differentiation and bone resorption activity, and the regulation of bone metabolic homeostasis mediated by macrophages has become one of the current research hotspots. Under specific stimuli, macrophages can polarise into the classically activated M1 phenotype with pro-inflammatory activity and the alternatively activated M2 phenotype with anti-inflammatory and reparative functions, and the imbalance in the M1/M2 polarisation ratio is one of the pathological factors contributing to the development and progression of osteoporosis (**Figure 1**); accumulating evidence indicates that abnormal shifts in macrophage polarisation phenotypes may disrupt the normal bone remodeling process and break the homeostatic balance of bone metabolism, therefore modulating the M1/M2 polarisation balance holds great significance for the treatment and prevention of osteoporosis, and the dynamic balance of M1/M2 macrophage polarisation also exerts a critical effect in various pathological conditions including tumours, cardiovascular diseases and immune disorders. This article systematically reviews the molecular mechanisms underlying the regulation of bone metabolism by macrophage polarisation, with a focus on summarising the advances in macrophage-targeted therapeutic strategies for osteoporosis, including the polarisation-regulating effects of conventional anti-osteoporotic drugs and the applications of emerging technologies such as nano delivery and cellular intervention, aiming to facilitate the development of more targeted and efficacious therapeutic strategies for osteoporosis based on macrophage polarisation and provide theoretical support and practical directions for addressing the public health challenge of osteoporosis against the backdrop of global ageing.

## 1 Introduction

Osteoporosis (OP) is a metabolic bone disease characterised by reduced bone tissue mass and degenerative changes in bone microarchitecture (1, 2), accompanied by decreased bone strength and increased fracture susceptibility, whose pathological hallmark is the uncoupling of bone resorption and bone formation homeostasis, involving multiple key factors such as impaired osteoblast function, excessive osteoclast activity and dysregulation of the osteoimmune microenvironment homeostasis (3, 4). A recent meta-analysis of the global prevalence of osteoporosis showed (5) that the global prevalence of osteoporosis is 18.3%, with 23.1% in women and 11.7% in men. Osteoporotic fractures most commonly occur in the vertebrae and hip, with a 1-year mortality rate of up to 20%-24% following hip fracture (6); osteoporotic fractures impose a heavy and increasing economic burden on global healthcare systems and societies, and it is estimated that the annual related costs in China will reach approximately US$19.92 billion by 2035 (7). With the intensification of population ageing, osteoporosis and its associated fractures have become increasingly prominent, seriously threatening the bone health of middle-aged and elderly people, especially postmenopausal women, and have become one of the major global public health issues (8).

The core pathological mechanism of osteoporosis is the imbalance of bone remodeling homeostasis (**Figure 2**). This pathological process is not driven by a single factor, but results from the interaction of complex factors including endocrine and metabolic disorders, chronic inflammation, deficiency of mechanical loading and osteoimmune imbalance (9). Endocrine factors play a crucial role in the pathogenesis of osteoporosis; in particular, the decline in oestrogen levels after menopause can promote the differentiation, maturation and survival of osteoclasts by regulating the RANK/RANKL/OPG signalling pathway, thereby significantly enhancing bone resorption (10). Meanwhile, abnormal parathyroid hormone levels, vitamin D deficiency and long-term glucocorticoid exposure can further exacerbate bone remodeling imbalance by inhibiting osteogenic activity, reducing bone matrix synthesis and promoting bone resorption (11). In addition, metabolic disorders are also an important link driving the progression of osteoporosis. With ageing, elevated oxidative stress levels, impaired mitochondrial function and altered bone marrow microenvironment can induce bone marrow mesenchymal stem cells to differentiate more into adipocytes rather than osteoblasts, leading to decreased osteogenic potential (12). In recent years, the role of inflammatory mechanisms in osteoporosis has received extensive attention. A state of chronic low-grade inflammation can induce sustained elevation of factors such as IL-1, IL-6 and TNF-α; these inflammatory mediators can not only directly promote osteoclastogenesis, but also amplify the bone resorption effect by regulating RANKL expression, thereby disrupting bone homeostasis (13). On the other hand, insufficient mechanical loading is also an important contributing factor to the development of osteoporosis. Long-term bed rest, immobilisation, weightlessness environment or lack of regular weight-bearing exercise can impair the perception and transduction of mechanical stimuli by osteocytes, downregulate osteogenic signals and upregulate sclerostin expression, resulting in decreased ability of the skeleton to undergo adaptive remodelling in response to external mechanical stimuli (14).

**Figure 1:**
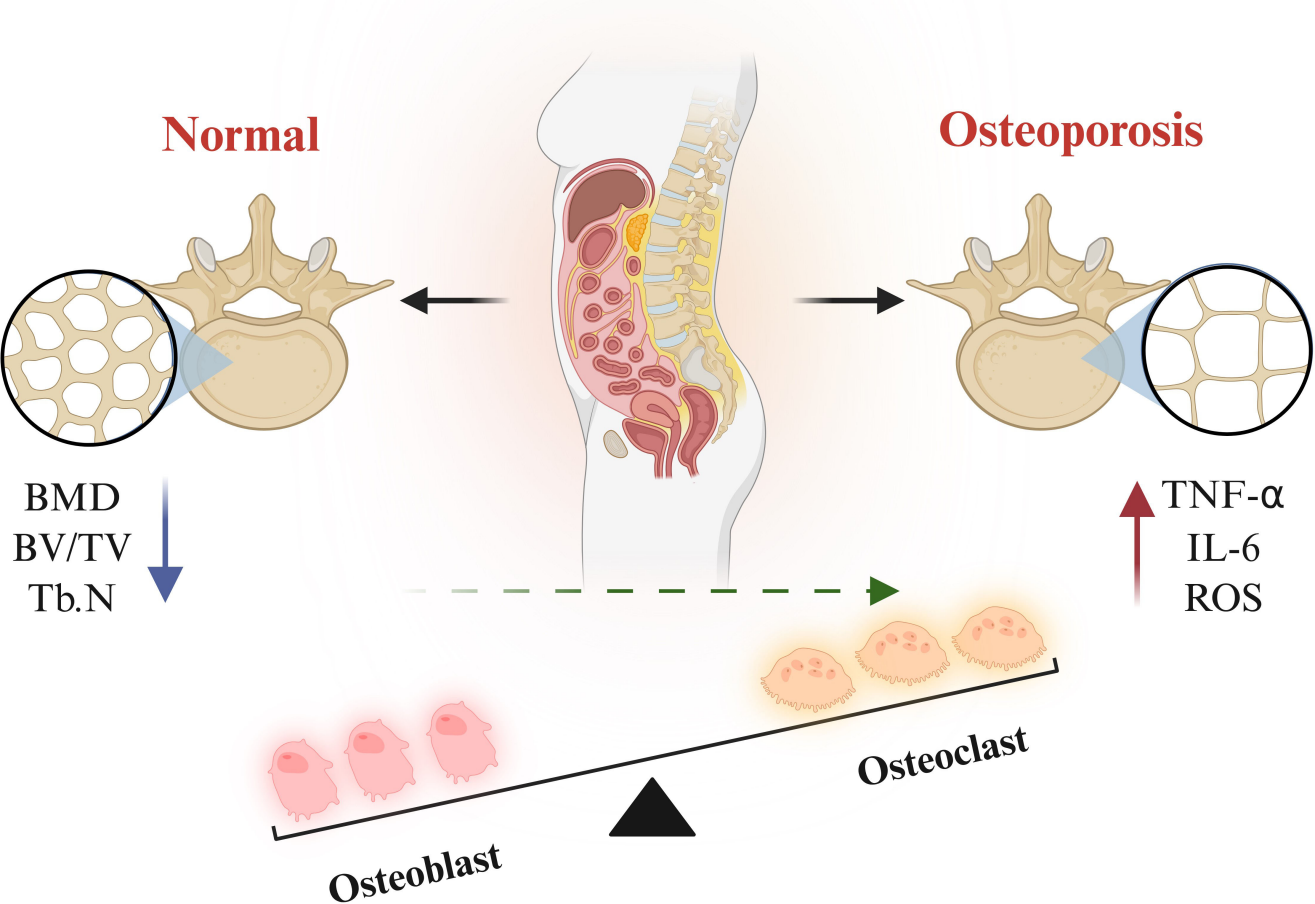
Imbalance in the M1/M2 polarisation ratio is one of the pathological factors contributing to the pathogenesis and progression of osteoporosis.

**Figure 2:**
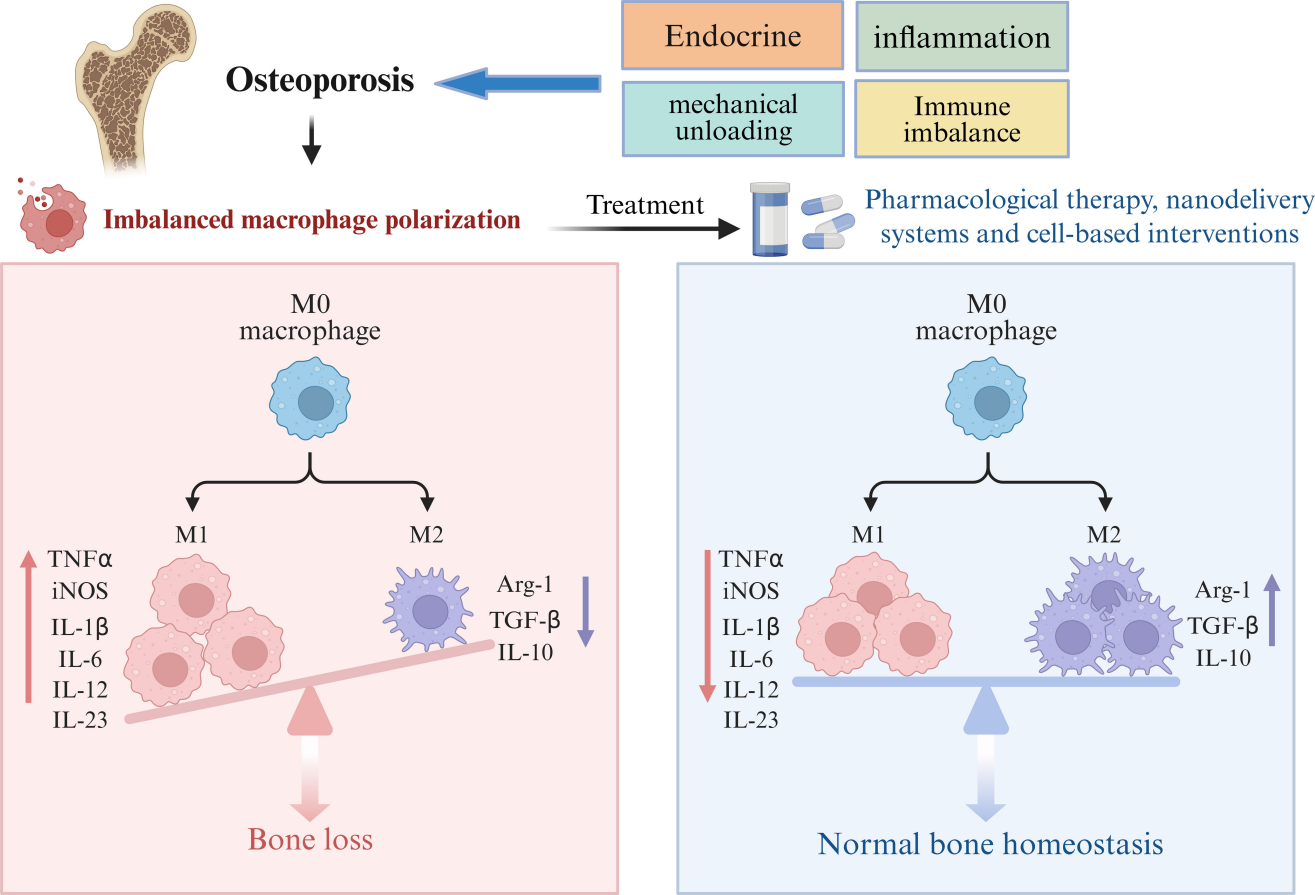
In osteoporosis, the functional balance between osteoblasts and osteoclasts is disrupted, manifested as enhanced osteoclast activity and impaired osteoblast function, which causes the rate of bone resorption to exceed that of bone formation, ultimately leading to bone mass loss and deterioration of bone microarchitecture.

Accumulating evidence in recent years has demonstrated (15, 16) that the maintenance of bone metabolic homeostasis depends not only on the reciprocal regulation between osteoblasts and osteoclasts, but also is closely related to the participation of the immune system. Among numerous immune cells, macrophages have attracted extensive attention due to their high plasticity and functional heterogeneity. As an important component of the bone marrow microenvironment, macrophages not only participate in inflammatory responses and the maintenance of tissue homeostasis, but also play a crucial role in bone remodelling by regulating osteoclast differentiation, affecting osteoblast function and participating in bone repair and regeneration processes (17, 18). In particular, macrophages in different polarisation states show significant differences in the regulation of bone metabolism; for example, pro-inflammatory M1 macrophages generally promote inflammatory responses and bone resorption, while anti-inflammatory and tissue repair-associated M2 macrophages contribute to bone formation and the restoration of bone homeostasis (19). Under osteoporosis-related pathological conditions such as ageing, oestrogen deficiency and chronic inflammation (20), the imbalance of macrophage polarisation can further disrupt bone metabolic homeostasis and accelerate disease progression. Macrophage polarisation, as a key target for regulating the osteoimmune microenvironment and bone metabolic balance, has demonstrated novel potential therapeutic value in the treatment of osteoporosis (21). Although the role of macrophages in osteoporosis has been widely recognised, many unknowns remain regarding its specific regulatory mechanisms: the specific molecular mechanisms of macrophage polarisation imbalance in different types of osteoporosis (postmenopausal, senile and diabetic osteoporosis), the precise interaction network between macrophages and other bone cells (osteoblasts, osteoclasts and bone marrow mesenchymal stem cells), and the therapeutic strategies targeting macrophage polarisation have not yet been fully elucidated. Therefore, this review will systematically summarise the mechanisms of action and therapeutic potential of macrophages in osteoporosis, providing a more microscopic perspective for understanding the pathological essence of osteoporosis and laying a foundation for the development of novel therapeutic strategies targeting the osteoimmune microenvironment.

## 2 Physiological regulation of bone metabolism by macrophage polarisation balance

### 2.1 Characteristics and functional differences of macrophage polarisation phenotypes

**Figure 3:**
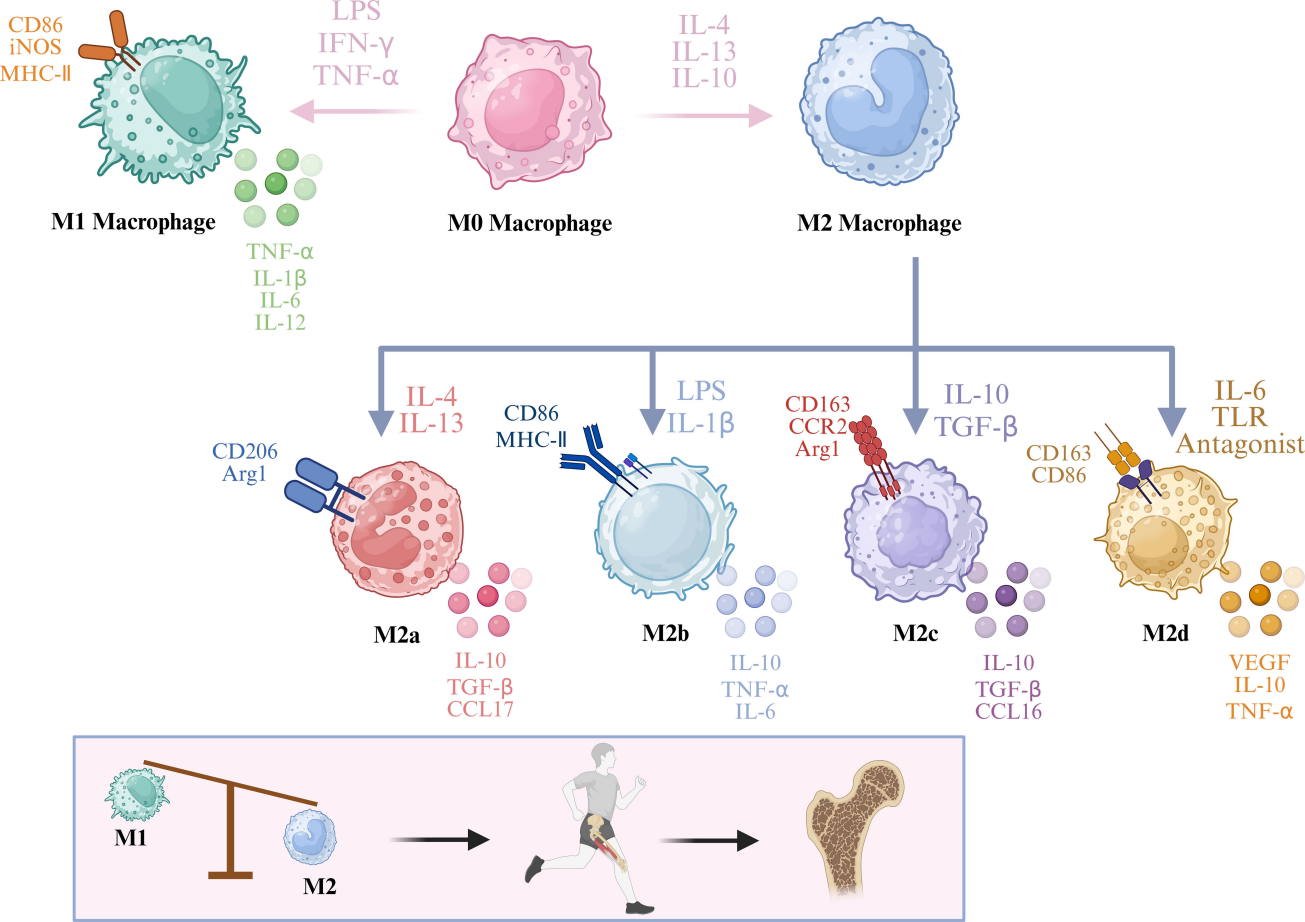
Imbalance of macrophage polarisation disrupts bone metabolic balance and leads to the development of osteoporosis.

Macrophages are the key effector cells in the osteoimmune microenvironment, and the high plasticity exhibited by their polarisation phenotypes is considered an important basis for maintaining and regulating bone metabolic homeostasis (22). According to different microenvironmental stimuli and functional characteristics, macrophages can be mainly divided into two functionally complementary but biologically distinct subpopulations: the M1 phenotype (classically activated) and the M2 phenotype (alternatively activated) (23, 24). The two subpopulations show significant differences in inducing signals, surface molecular markers, cytokine secretion profiles and regulatory effects on bone metabolism (**Figure 3**). They undertake distinct functions of inflammation initiation and tissue repair respectively during the bone remodelling process, and together constitute a dynamically coordinated “initiation-repair” regulatory axis in the bone remodelling cycle.

#### 2.1.1 M1 macrophages

As a pro-inflammatory polarisation subpopulation, M1 macrophages play an important regulatory role in bone metabolic imbalance during the pathogenesis and progression of osteoporosis (25). Their activation is mainly dependent on exogenous or endogenous stimulatory signals such as lipopolysaccharide (LPS), interferon-γ (IFN-γ) and tumour necrosis factor-α (TNF-α) (26, 27). They participate in the regulation of bone metabolic homeostasis by initiating innate immune responses, and their main functions are to initiate bone resorption, eliminate pathogens and regulate local inflammation (28). In terms of molecular phenotypic characteristics, M1 macrophages exhibit typical expression profiles of pro-inflammatory markers (29). In terms of surface molecular markers, M1 macrophages generally highly express the co-stimulatory molecule CD86, the myeloid cell activation-associated marker CD11c, and major histocompatibility complex class II (MHC-II), a key molecule for antigen presentation (30–32). Among them, CD86 can enhance immune responses by binding to CD28 on the surface of T cells (33), while MHC-II mainly mediates antigen presentation and participates in the activation of adaptive immunity, thereby providing an important molecular basis for signal transduction along the “bone-immune” axis in the bone microenvironment. At the intracellular functional molecular level, M1 macrophages significantly upregulate the expression of inducible nitric oxide synthase (iNOS) and promote the production of nitric oxide (NO), thereby enhancing their ability to clear damaged tissues and pathogen-associated stimuli (34, 35). In addition, they also show high expression of genes encoding various pro-inflammatory cytokines such as TNF-α and interleukin-1β (IL-1β) (36), which ensures that they can respond rapidly and effectively to inflammatory signals related to the initiation of bone resorption.

In terms of secretory function, M1 macrophages exhibit a secretory profile characterised by pro-inflammatory and pro-matrix-degradation properties, and the various cytokines, proteases and chemokines they release play a crucial role in bone remodelling imbalance. On the one hand, TNF-α and IL-1β secreted by M1 macrophages are key upstream signals for osteoclast differentiation (37). TNF-α binds to TNFR1 on the surface of osteoclast precursor cells, activates the downstream NF-κB pathway and promotes the nuclear translocation of NFATc1, the key transcription factor for osteoclast differentiation, while IL-1β enhances the transcriptional activity of NFATc1 by activating the MAPK pathway. The two exert a synergistic effect to promote osteoclast formation and significantly strengthen the efficiency of bone resorption initiation (38). On the other hand, M1 macrophages can secrete matrix metalloproteinases (MMP-9, MMP-12) and chemokines (CXCL9, CXCL10) (39, 40). Among them, MMP-9 specifically degrades type I collagen fibres in the bone matrix, disrupts the structural integrity of the bone matrix and provides physical space for the migration and adhesion of osteoclasts and the formation of bone resorption lacunae (41), while CXCL9 and CXCL10 recruit more osteoclast precursor cells and immune cells to bone remodelling sites through chemotactic effects, amplifying the early bone resorption initiation effect (42).

#### 2.1.2 M2 macrophages

M2 macrophages are an important functional subpopulation that mediates anti-inflammatory responses and tissue repair in the osteoimmune network, and play a critical role in maintaining the homeostasis of the bone tissue microenvironment and promoting regeneration after injury. Existing studies have shown (43) that monocytes tend to differentiate into M2 macrophages under the induction of macrophage colony-stimulating factor (M-CSF), while they are more likely to differentiate into M1 macrophages under the action of granulocyte-macrophage colony-stimulating factor (GM-CSF). The activation of M2 macrophages is mainly dependent on anti-inflammatory-related signalling molecules such as IL-4, IL-13 and transforming growth factor-β (TGF-β) (44). Functionally, M2 macrophages not only have significant anti-inflammatory and immunomodulatory effects, but also can promote angiogenesis, tissue repair and regenerative reconstruction at the injury site (45, 46), thus being regarded as important regulatory cells in the process of bone repair and bone regeneration. Notably, the polarisation state of macrophages is dynamically regulated by multiple cytokines in the local microenvironment. According to different inducing factors and functional characteristics, M2 macrophages can be further divided into different subtypes including M2a, M2b, M2c and M2d (47). The inducing factors and main biological functions of each subtype are shown in **Table 1**.

**Table 1:** Macrophage phenotypes and their respective inducing factors, markers, secreted cytokines and biological functions.

Polarization phenotype	Inducing factor	Marker	Secreted cytokines	Biological functions
M1 type	LPS、IFN-γ、TNF-α	CD86、CD11c、MHC-II、iNOS	TNF-α、IL-1β、IL-6、IL-12、MMP-9、MMP-12	clearing pathogens and necrotic tissues (26, 27)
M2a type	IL-4、IL-13	CD206、Arg1	IL-10、TGF-β、CCL17、CCL18、CCL22	promoting bone matrix mineralization and tissue repair (48)
M2b type	Immune Complex、LPS、IL-1β	CD86、MHC-II	IL-10、TNF-α、IL-6、CCL1、CCL3	regulating T cell activation and modulating immune homeostasis (49)
M2c type	IL-10、TGF-β	CD163、CCR2、Arg1	IL-10、TGF-β、CCL16、CCL18	phagocytosing apoptotic cells and reducing the release of inflammatory factors (50)
M2d type	Adenosine、IL-6、TLR Antagonist	CD163、CD86	VEGF、IL-10、TNF-α	promoting angiogenesis and tumor growth (51–53)

Studies have shown (54–56) that M2 macrophages highly express molecules such as the mannose receptor (MR/CD206), the scavenger receptor CD163 and the chemokine receptor CCR2. Among them, CD206 participates in the phagocytic clearance of damaged tissues and cell debris by recognising mannose residues in the bone matrix, thereby promoting the local repair process, while CD163 reduces oxidative stress damage by clearing free hemoglobin-haptoglobin complexes in the blood (57), providing a guarantee for the homeostasis of the bone microenvironment. In addition, M2 macrophages significantly upregulate the expression of arginase 1 (Arg1), which competes with iNOS in M1 macrophages for arginine utilisation, and promotes extracellular matrix synthesis, tissue repair and wound healing by generating polyamines and proline (58, 59). In terms of secretory profile, M2 macrophages highly express a variety of cytokines and growth factors closely related to anti-inflammatory repair, such as IL-10, TGF-β and BMP-2 (60). In an *in vitro* study (61), researchers isolated BMSCs from fresh mouse femurs and found that treatment with TGF-β1 increased the mRNA and protein expression of the transcriptional co-activator TAZ, while enhancing Runx2-dependent transcription and inhibiting PPARγ2-dependent transcription. Another study found (62) that TGF-β2 inhibited PPARγ2 expression accompanied by increased Runx2 expression in bone marrow stromal cells from a rat hindlimb unloading model, thereby suppressing adipogenic differentiation. BMP-2 can bind to BMPR-IA/IB on the surface of osteoblasts, activate the Smad1/5 pathway and promote its nuclear translocation, upregulate the expression of Runx2, a key transcription factor for osteogenic differentiation, and osteocalcin (OCN) (63), significantly enhancing bone matrix synthesis and mineralisation capacity. In summary, M2 macrophages jointly promote inflammation resolution, tissue remodelling and bone regeneration after bone tissue injury through surface receptor-mediated clearance and repair functions, arginine metabolic reprogramming, and the secretion of various anti-inflammatory and osteogenic factors.

#### 2.1.3 Dynamic plasticity of macrophage polarisation states

Chronic inflammation is a direct pathogenic factor inducing osteoporosis (64), and relevant clinical data confirm that patients with osteoporosis have elevated serum levels of inflammatory factors such as TNF-α and IL-6 (65). M1 macrophages are the producers of these inflammatory factors, which clear pathogens and necrotic tissues by releasing ROS, NO and pro-inflammatory cytokines. Therefore, prolonged activity of M1 macrophages may lead to tissue damage and inflammation-related diseases (66, 67). An elevated M1/M2 ratio has been confirmed to be involved in the underlying pathogenic mechanisms of chronic inflammatory diseases (68, 69). For example, in obesity-associated insulin resistance, increased infiltration of M1 macrophages in adipose tissue leads to secretion of IL-6 and TNF-α, which can inhibit the insulin receptor signalling pathway in adipocytes and reduce insulin sensitivity (70). In the process of diabetic wound healing, macrophages are key regulatory factors, and their reduced ability to switch from the inflammation-associated M1 phenotype to the pro-healing M2 phenotype ultimately leads to delayed wound closure or even amputation (71).

Macrophages possess a high degree of phenotypic continuum and plasticity (72), and their polarisation states are not static, discrete categories but exist in a dynamic process regulated by the microenvironment (73). Under the influence of multiple factors including inflammatory mediators, growth factors, metabolic signals and intercellular interactions (74), macrophages can flexibly transition between different polarisation states along a continuous functional spectrum, thereby adapting to various physiological and pathological demands such as maintenance of tissue homeostasis, inflammatory responses and repair remodelling (75). Relevant studies have found (76) that the M1/M2 ratio is increased in the bone marrow of ovariectomised (OVX) osteoporotic mice, and it has been proposed that an elevated M1/M2 ratio may lead to enhanced osteoclastogenesis. In addition, an increase in M1 macrophages impairs the activity of the tricarboxylic acid (TCA) cycle and mitochondrial oxidative phosphorylation (OXPHOS), leading to the accumulation of TCA cycle metabolites such as succinate and citrate, which in turn increases the production of lactate and ROS (77, 78). Conversely, Arg1 expressed by M2 macrophages can provide substrates for collagen synthesis by producing ornithine, thereby promoting bone tissue repair and matrix maturation, and its reduction may inhibit this process (79, 80).

However, polarisation imbalance does not merely manifest as excessive M1 activation and relative M2 deficiency. In the early stages of bone injury or infection, an appropriate and transient M1 response is crucial for clearing pathogens and necrotic tissues, recruiting bone marrow mesenchymal stem cells and initiating repair signals (81, 82). Insufficient or prematurely inhibited M1 activation may lead to inadequate debridement, attenuated initial inflammatory signals and insufficient recruitment of subsequent repair cells, thereby delaying bone healing (83). Meanwhile, M1 macrophages transiently accumulate in the early stage of bone resorption; after initiating bone resorption, the MMP-9 they secrete will release osteogenic factors such as TGF-β and BMPs stored in the matrix when degrading the bone matrix (84). These factors can further recruit bone marrow mesenchymal stem cells and induce the conversion of M1 to M2 macrophages, providing signal reserves for the initiation of the subsequent bone formation phase (85). On the other hand, although the M2 phenotype is beneficial for inflammation resolution and tissue repair, its excessive activation or persistent dominance may also lead to pathological consequences, such as promoting fibrosis, abnormal tissue remodelling, and even causing insufficient ossification of the repaired tissue, resulting in the formation of a fibrous callus rather than a bony callus (86). In specific pathological microenvironments, excessive M2-like macrophages may also participate in immunosuppression and disease progression associated with bone marrow fibrosis and tumour bone metastasis (87, 88). Therefore, therapeutic strategies targeting macrophage polarisation in osteoporosis should not simply inhibit M1 and promote M2, but reconstruct a temporal dynamic balance matched to the stage of the disease course.

### 2.2 Interactions between macrophages and bone cells in osteoimmunology

Osteoimmunology is an interdisciplinary discipline that studies the interactions between the skeletal system and the immune system. The concept of “osteoimmunology” was formally proposed in 1997 (89), which broke through the traditional view that the regulation of bone homeostasis mainly depends on bone cells and promoted the gradual development of this field into an independent research direction. Osteoimmunology plays an important role in maintaining the dynamic balance of bone metabolism and the structural stability of bone tissue, and is an important mechanism ensuring the normal morphology and function of the skeleton. Accumulating studies have shown (90–92) that osteoimmune imbalance is closely related to the pathogenesis and progression of various bone-related diseases such as osteoporosis, rheumatoid arthritis and bone tumours, and is an important pathological basis for these diseases. In this process, macrophages participate in the regulation of bone remodelling through the dynamic transition of different polarisation phenotypes and play an important role in maintaining the homeostasis of the bone microenvironment.

#### 2.2.1 Macrophages and osteoclasts

Both macrophages and osteoclasts are derived from monocyte-macrophage lineage precursor cells differentiated from bone marrow hematopoietic stem cells, but can differentiate into cell types with distinct biological functions under the induction of different microenvironmental signals (93, 94). Macrophages are widely distributed in almost all tissues, among which bone-resident macrophages, the resident macrophages in bone tissue, account for approximately 15% to 20% of the total bone marrow cells in mice (95, 96). Existing studies have shown (97) that in an *in vitro* culture model of bone-resident macrophages derived from neonatal mice, continuous stimulation with RANKL can induce their differentiation into TRAP-positive osteoclasts with bone resorption capacity. As an important component of the immune system, M1 macrophages mainly participate in inflammatory responses and host defence by secreting pro-inflammatory cytokines. Studies have shown (98) that high levels of inflammatory cytokines can promote the differentiation and maturation of osteoclasts, thereby enhancing bone matrix resorption. For example, IL-1 can not only upregulate the expression of RANKL in stromal cells, but also directly promote the differentiation of osteoclast precursor cells, thus mediating TNF-induced osteoclastogenesis (99). In contrast, M2 macrophages mainly inhibit the formation and activity of osteoclasts by secreting anti-inflammatory cytokines, promoting the synthesis of OPG and downregulating the expression of RANKL (100). Therefore, the polarisation state of macrophages is of great significance in osteoclast differentiation and the regulation of bone metabolism.

#### 2.2.2 Macrophages and osteoblasts

**Figure 4:**
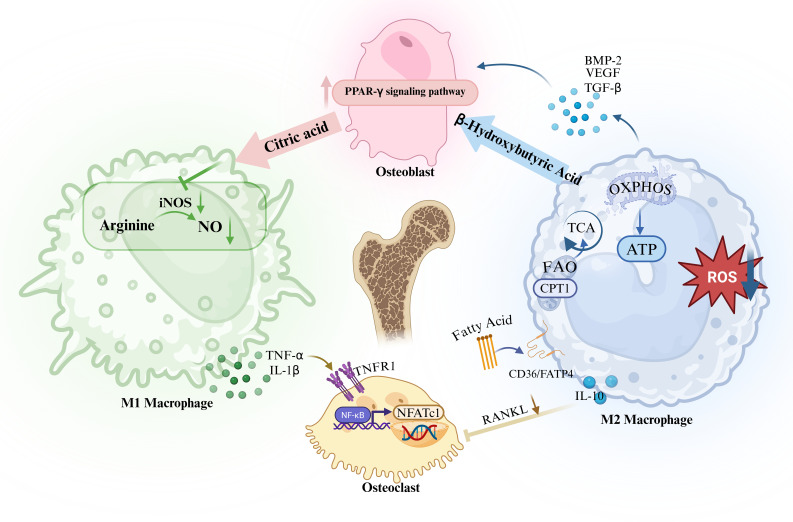
M1 macrophages promote osteoclast differentiation and maturation and enhance bone resorption by secreting pro-inflammatory factors such as TNF-α and IL-1β; M2 macrophages inhibit excessive bone resorption by secreting anti-inflammatory and osteogenic factors such as IL-10 and TGF-β, while promoting osteoblast function and bone formation.

In the process of maintaining bone homeostasis, macrophages not only exhibit significant pro-inflammatory effects in inflammatory bone diseases, but also play an important regulatory role in bone mass accumulation and bone tissue repair. Osteoblasts, as the effector cells of bone formation and bone metabolic balance, are mainly responsible for the synthesis, secretion and mineralisation of bone matrix (101). Accumulating studies have shown (102, 103) that macrophages, especially M2 macrophages, can promote osteoblast differentiation and enhance osteogenic activity by secreting cytokines such as BMP-2, VEGF and TGF-β. Studies have shown (104) that macrophage-deficient mice have a decrease in bone mineral density of approximately 25% and a reduction in trabecular number of approximately 70%; while depletion of macrophages during fracture repair significantly impairs bone healing, further suggesting the critical role of macrophages in bone formation and bone repair. Notably, the interaction between macrophages and osteoblasts is also finely regulated by the metabolic state of the bone microenvironment. On the one hand, M2 macrophages mainly rely on oxidative phosphorylation and fatty acid oxidation for energy supply (105), and ketone bodies produced during their metabolism, such as β-hydroxybutyrate, may promote bone matrix mineralisation by activating the PPAR-γ-related pathway in osteoblasts; on the other hand, bone matrix metabolites secreted by osteoblasts, such as citrate, can inhibit macrophage iNOS activity (106) and reduce NO production, thereby indirectly inhibiting M1 macrophage polarisation and maintaining the metabolic homeostasis of the bone microenvironment (**Figure 4**). Therefore, macrophages play an important role in maintaining bone homeostasis and promoting fracture repair by regulating osteoblast differentiation and function.

#### 2.2.3 Macrophages and BMSCs

Bone marrow mesenchymal stem cells (BMSCs) are important functional cells for bone tissue repair and regeneration, which can differentiate into multiple lineages including osteoblasts, chondrocytes and adipocytes (107). Accumulating studies have shown (108) that the crosstalk between BMSCs and macrophages runs through the entire process of bone repair, bone remodelling and immune homeostasis maintenance, and their functional imbalance is an important pathological basis for diseases such as osteoporosis and non-union of bone defects. Exosomes, as an important component of extracellular vesicles, are rich in functional molecules such as miRNAs, proteins and lipids, and are important mediators of intercellular information transmission (109). Existing evidence suggests that exosomes are one of the key carriers for communication between macrophages and BMSCs (110). For example, in *in vivo* studies, exosomes derived from M2 macrophages (M2-Exos) can promote osteogenic differentiation of BMSCs and inhibit the differentiation of bone marrow-derived macrophages into osteoclasts, thereby alleviating pathological alveolar bone resorption (111); in *in vitro* and related animal models, M2-Exos can also enhance tendon-bone healing potential by attenuating BMSC senescence (112). Conversely, BMSCs can also induce macrophage polarisation towards the M2 phenotype by secreting exosomes to maintain the homeostasis of the osteoimmune microenvironment. Studies have shown (113) that BMSC-derived exosomes (BMSCs-Exos) can alleviate macrophage-driven inflammation, induce macrophage differentiation into the anti-inflammatory M2 phenotype and inhibit osteoclast formation. In addition, specific miRNAs carried by BMSCs-Exos are also involved in this process, among which relevant miRNAs are considered to exert bone protective effects by downregulating pro-inflammatory cytokine levels, reducing inflammatory cell infiltration and promoting M2 polarisation (114, 115).

### 2.3 Signalling pathways regulating macrophage polarisation balance

In the process of maintaining bone homeostasis, the balance of macrophage polarisation is an important basis for regulating the osteoimmune microenvironment and the dynamic coupling of bone formation and bone resorption. Existing studies have shown (116) that macrophage polarisation is not driven by a single signal, but is co-regulated by multiple intersecting signalling pathways, among which NF-κB, JAK/STAT and PI3K/AKT are the core pathways involved in this process. These signalling pathways can integrate multiple stimuli such as inflammatory cytokines, metabolic signals and intercellular communication to determine the transformation of macrophages into different functional phenotypes. Therefore, elucidating the key signalling pathways that regulate macrophage polarisation balance in osteoporosis will not only help deepen the understanding of the regulatory mechanisms of osteoimmunology, but also provide a theoretical basis for identifying potential intervention targets.

#### 2.3.1 NF-κB signalling pathway

During the pathogenesis and progression of osteoporosis, aberrant activation of the NF-κB signalling pathway and macrophage polarisation imbalance mutually reinforce each other, forming a vicious cycle, which is considered an important mechanism that disrupts bone metabolic homeostasis and accelerates bone mass loss (117). Under normal physiological conditions, the NF-κB signalling pathway can respond to changes in the local microenvironment and moderately regulate macrophage polarisation, thereby maintaining the dynamic balance between M1 and M2 macrophages. However, in the osteoporotic state, massive accumulation of pro-inflammatory factors such as TNF-α and IL-1β continuously stimulates macrophages, leading to sustained and excessive activation of the NF-κB signalling pathway, which in turn induces a pathological shift of macrophage polarisation towards the pro-inflammatory phenotype.

The NF-κB signalling pathway mediates the expression of multiple inflammatory factors in the innate immune system (118). Therefore, inhibiting the expression of the NF-κB signalling-dependent NLRP3 inflammasome in macrophages can reduce the release of inflammatory cytokines (119). Excessively activated NF-κB pathway promotes the phosphorylation and nuclear translocation of the p65/p50 dimer (120), significantly upregulates the expression of M1-related genes such as iNOS and TNF-α, and leads to an increased proportion of M1 macrophages. The increased number of M1 macrophages, on the one hand, enhances osteoclast activity and accelerates bone resorption by secreting a large number of pro-inflammatory factors; on the other hand, inhibits the proliferation and differentiation of osteoblasts and weakens the compensatory capacity of bone formation. Meanwhile, the persistently activated NF-κB pathway can also inhibit the differentiation and maturation of M2 macrophages by affecting the activity and nuclear translocation of STAT6, the key transcription factor for M2 polarisation (121), resulting in reduced secretion of osteogenic factors and further exacerbating bone homeostasis imbalance.

In addition, multiple studies have shown (122) that CX3CL1 is involved in the pathological process of osteoporosis. Upregulated CX3CL1 promotes M1 macrophage polarisation and osteoclast differentiation by activating the NF-κB signalling pathway, while inhibition of this signalling pathway can alleviate inflammatory responses and bone loss in osteoporotic mouse models (123). However, such studies also suggest that although interventions targeting a single inflammatory axis can achieve certain effects under experimental conditions, whether they can achieve stable and long-lasting bone protective effects in clinical practice remains to be verified by more high-quality studies. Overall, targeted regulation of the NF-κB signalling pathway and restoration of macrophage polarisation balance may provide new intervention ideas for the prevention and treatment of osteoporosis, but its clinical translation value needs to be further clarified.

#### 2.3.2 JAK/STAT signalling pathway

The JAK/STAT signalling pathway is an important pathway controlling immune cell homeostasis (124), and numerous studies have reported that macrophage phenotype and activation are regulated by JAK/STAT signal transduction. Existing studies have confirmed (125, 126) that the JAK/STAT pathway is involved in the progression of various inflammatory diseases, and inhibition of its activation can regulate macrophage polarisation and effectively reduce related inflammatory damage. Among them, synovial inflammation is an important driving factor for the progression of temporomandibular joint osteoarthritis. Synovial M1 macrophages induce synovitis by secreting inflammatory mediators and MMPs, thereby accelerating the process of cartilage tissue degradation and skeletal structural degeneration (127). Therefore, relevant studies have shown that inhibiting the activation of the JAK2/STAT3 pathway can reduce M1 macrophage infiltration and enhance M2 macrophage polarisation, and inhibit the expression of inflammatory markers including TNF-α, IL-6 and iNOS, thereby reducing synovial inflammation and cartilage degradation (128).

In macrophages, LPS can induce elevated levels of inflammatory cytokines, which activates receptor-associated JAK kinases. Activated JAK further promotes the phosphorylation of STAT proteins, and phosphorylated STATs form homo- or heterodimers in the cytoplasm, which are then translocated to the nucleus to regulate the expression of inflammatory mediators as transcription factors (129, 130). In summary, during excessive inflammatory responses, inhibiting JAK/STAT activation through intervention measures is crucial for controlling the inflammatory process and maintaining body homeostasis. The suppressor of cytokine signalling (SOCS) family acts as negative regulators of the JAK/STAT pathway (131). Studies have found (132) that decreased expression of SOCS1 activates the JAK1/STAT1 pathway, thereby promoting macrophage polarisation towards the M1 phenotype. Phosphorylation of STAT3 plays an important role in macrophage polarisation towards the M2 phenotype. When the expression level of phosphorylated STAT3 protein in the JAK/STAT pathway is upregulated, it can regulate macrophage polarisation towards the M2 phenotype. This process can accelerate the migration rate of osteoblasts and upregulate the expression of osteogenesis-related proteins BMP-2, ALP and RUNX2, while recruiting more M2 macrophages to the bone defect area, thereby promoting early bone formation (133).

#### 2.3.3 PI3K/AKT signalling pathway

The PI3K/AKT pathway and its downstream targets have become central regulators of macrophage activation phenotypes (134), and also participate in coordinating macrophage responses to different metabolic and inflammatory signals. Activation of PI3K has been confirmed to induce the activation of M2 macrophages (135), while upregulating the activity of Arg1 in SHIP-deficient macrophages, thereby exerting anti-inflammatory and repair effects. However, PTEN, as a negative regulator of the PI3K/AKT pathway, its deletion can lead to increased AKT activity accompanied by inhibition of LPS responses in macrophages, which contributes to the shift of macrophages towards the M2 phenotype (136). Therefore, targeting PTEN may become an important strategy for balancing macrophage polarisation. The TSC/mTORC1 pathway, as a downstream effector of PI3K/AKT, is also involved in the regulation of macrophage polarisation. In TSC1-deficient macrophages, AKT activity is significantly downregulated, which increases inflammatory responses and reduces the production of anti-inflammatory cytokines (137). Therefore, relevant studies have shown that the decreased AKT activity after TSC1 deletion in macrophages may be due to mTORC1-mediated negative feedback of AKT signalling (138). In ovariectomised animal models of postmenopausal osteoporosis and corresponding *in vitro* experiments (139), activation of the PI3K/AKT signalling pathway can promote the differentiation of bone marrow mesenchymal stem cells into osteoblasts and enhance osteoblast survival, thereby improving bone formation capacity and reducing bone loss. Recent studies have found (140) that exosomal miR-125b-5p from macrophages targets IGF2 and significantly inhibits the activation of the PI3K/AKT pathway, leading to decreased expression of key osteogenic markers such as Col1A, Runx2 and OPN. Knockdown of miR-125b-5p can restore PI3K/AKT activity and increase bone mineral density.

## 3 Molecular mechanisms of macrophage polarisation imbalance in osteoporosis

**Figure 5:**
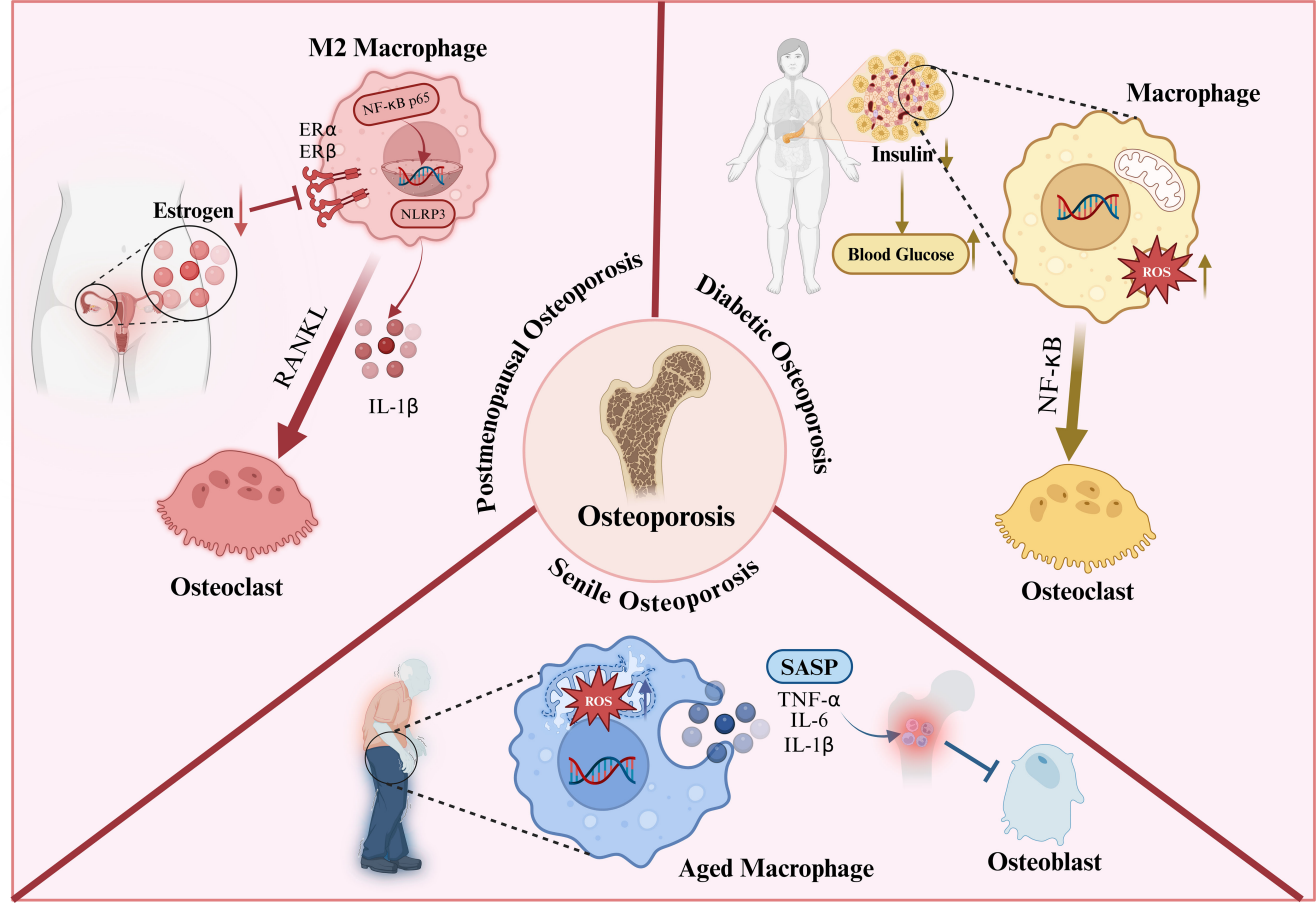
Oestrogen deficiency, ageing and high-glucose environment can all lead to macrophage functional imbalance, disrupt the osteoimmune microenvironment and bone metabolic homeostasis, and thereby promote the onset and progression of osteoporosis.

Osteoporosis is a metabolic bone disease characterised by increased fracture risk, which severely impairs the quality of life of the elderly population. Recent studies have shown that macrophages, as important regulatory cells in the osteoimmune microenvironment, participate in the regulation of bone metabolism by secreting a variety of cytokines and inflammatory mediators. Among them, M1 macrophages mainly exhibit pro-inflammatory and pro-osteoclast effects, while M2 macrophages are more associated with anti-inflammatory responses, tissue repair and osteogenic processes. Therefore, the imbalance of macrophage polarisation states is considered one of the important mechanisms underlying the pathogenesis and progression of osteoporosis. This section will summarise the molecular mechanisms and differences of macrophage polarisation imbalance in different types of osteoporosis (**Figure 5**).

### 3.1 Postmenopausal osteoporosis

Postmenopausal osteoporosis (PMOP) is a metabolic bone disease caused by ovarian function decline and a sharp drop in oestrogen levels in women after menopause. Oestrogen inhibits the differentiation of osteoclast precursors into mature osteoclasts, accelerates the apoptosis of mature osteoclasts and reduces bone resorption (141); it also promotes the synthesis of type I collagen by osteoblasts, regulates the deposition of minerals such as calcium and phosphorus in the bone matrix, and improves the mechanical strength of bone. Studies have found that bone marrow macrophages in OVX model mice show increased M1 polarisation and impaired M2 polarisation, which can be reversed by oestrogen intervention treatment (142). Therefore, macrophage polarisation imbalance caused by postmenopausal oestrogen deficiency may be an important molecular pathological basis driving the development of PMOP.

In recent years, the relationship between macrophage polarisation and oestrogen levels has attracted much attention in PMOP. Macrophages are important target cells of oestrogen, and they express two key oestrogen receptors, ERα and ERβ, on their cell membrane, in the cytoplasm and nucleus (143), which are important factors for oestrogen to regulate the balance of macrophage polarisation. Existing studies have shown (144) that in OVX models of postmenopausal osteoporosis, oestrogen deficiency leads to an increased M1/M2 ratio in bone marrow macrophages, while 17β-oestradiol mainly inhibits the nuclear translocation of NF-κB p65 through ERα and enhances anti-inflammatory signals such as IL-10, thereby promoting the shift of macrophages towards the M2-like phenotype, reducing pro-osteoclastogenic changes and alleviating bone loss. Clinical studies have found (145) that M2 activation appears to be attenuated in macrophages from postmenopausal women, and oestrogen treatment prevented the inhibitory effect of LPS/IFN-γ on the expression of human M2 macrophage markers and cytokines, suggesting that menopausal oestrogen deficiency is closely associated with impaired M2 polarisation. In addition, oestrogen can exert a selective protective effect on M2 macrophages under RANKL stimulation. Relevant studies have shown (76) that oestrogen, mediated by ERα, blocks the downstream signals of NF-κB p65 nuclear translocation and selectively protects M2 macrophages from RANKL stimulation; in the absence of oestrogen, M2 macrophages differentiate into functional osteoclasts under RANKL stimulation. Thus, in the context of decreased oestrogen levels, macrophages may play a key driving role in the pathogenesis and progression of PMOP by directly promoting the differentiation and maturation of osteoclasts. On the other hand, oestrogen deficiency in postmenopausal women leads to excessive activation of inflammatory cytokines. Under chronic inflammatory conditions, the NLRP3 inflammasome is persistently activated, releasing large amounts of mature IL-1β. In the regulatory mechanism of bone metabolism, IL-1β is recognised as the main mediator (146). Secreted by macrophages, IL-1β binds to its receptor and accelerates the production of RANKL, thereby excessively activating osteoclast formation (147).

### 3.2 Senile osteoporosis

Senile osteoporosis (SOP) is a metabolic bone disease closely related to organismal ageing (148), which mostly occurs in the elderly population aged 65 years and above. Its main pathological characteristics are continuous bone loss, gradual degeneration of bone microarchitecture and decreased bone strength with ageing, ultimately leading to increased bone fragility and significantly elevated fracture risk. With the progression of ageing, senescent cells accumulate continuously in the bone microenvironment, and clearing senescent cells in bone tissue has been proven to delay age-related bone loss to a certain extent (149). Recent studies have shown that senescent macrophages play an important role in the occurrence and development of SOP. Senescent macrophages accumulated in the bone marrow can secrete granulocalcin (150), promote the differentiation of bone marrow mesenchymal stem cells into adipocytes, and inhibit osteoblast formation at the same time, thereby accelerating bone marrow adipogenesis and impaired bone formation, and ultimately driving the process of skeletal ageing and osteoporosis.

Senescent macrophages can secrete a variety of senescence-associated secretory phenotype (SASP) factors, such as tumour necrosis factor-α (TNF-α), interleukin-6 (IL-6) and interleukin-1β (IL-1β) (151). These pro-inflammatory mediators reshape the inflammatory status of the bone microenvironment, leading to the development of chronic low-grade inflammation associated with organismal ageing. This persistent inflammatory microenvironment not only disrupts the crosstalk between macrophages and bone cells, but also contributes to the onset and progression of senile osteoporosis (SOP) by promoting osteoclastogenesis and inhibiting osteogenesis. Relevant studies have demonstrated (152) that the expression of M1 macrophage markers (CD68, TNF-α and IL-6) is elevated in aged mice, whereas the expression of M2 markers (CD36 and interleukin-10 (IL-10)) is decreased, suggesting that the polarisation tendency of macrophages shifts from an anti-inflammatory reparative phenotype to a pro-inflammatory phenotype during the ageing process; this immune imbalance characterised by enhanced M1 polarisation further promotes the expression of senescence-associated molecules and accelerates bone tissue degeneration. Furthermore, mitochondria, as the core organelles governing cellular energy metabolism and homeostasis maintenance, their dysfunction is also one of the key mechanisms by which senescent macrophages drive bone metabolic disorders (153). Studies have found (154) that the ageing process induces mitochondrial dysfunction in bone tissue macrophages, including reduced mitochondrial number, decreased membrane potential and elevated levels of reactive oxygen species (ROS). These changes not only augment the pro-inflammatory response of macrophages, but may also alter the metabolic profile of bone marrow mesenchymal stem cells (BMSCs) - manifesting as impaired aerobic respiration and enhanced glycolysis - thereby inhibiting the expression of osteogenic differentiation-related genes and promoting their adipogenic differentiation. Taken together, senescent macrophages participate in the pathological process of SOP through multiple convergent pathways: SASP release, enhanced pro-inflammatory polarisation and mitochondrial dysfunction, hence inhibiting the excessive polarisation of macrophages towards the M1 phenotype and delaying their senescence may represent a promising strategy for intervening in age-related bone loss and bone microenvironment imbalance.

### 3.3 Diabetic osteoporosis

Diabetic osteoporosis (DOP) is one of the common complications in patients with diabetes mellitus, which is mainly caused by abnormal bone metabolism secondary to glycemic metabolic disorder, leading to increased fracture risk and delayed fracture healing. Relevant studies have shown (155, 156) that DOP is associated with hyperglycemia, calcium and phosphorus metabolic disorder and insulin deficiency. Long-term hyperglycemia modifies bone matrix proteins through non-enzymatic glycation reactions, impairing the structural stability and mechanical strength of the bone matrix. Meanwhile, osmotic diuresis induced by hyperglycemia increases urinary calcium excretion, resulting in systemic negative calcium balance and indirectly promoting bone resorption.

Immune homeostasis imbalance is one of the key pathological features of DOP, which is intrinsically closely associated with glycemic metabolic disorder, oxidative stress and abnormal cellular energy metabolism (157). A high-glucose environment can significantly elevate intracellular reactive oxygen species (ROS) levels in macrophages, induce oxidative stress responses, and thereby lead to macrophage polarisation imbalance and abnormal immune regulation (158). Mechanistically, excessive ROS promotes the polarisation of macrophages towards the M1 pro-inflammatory phenotype and exacerbates local inflammatory responses by activating inflammation-related signalling pathways such as NF-κB. Meanwhile, sustained hyperglycemia can further amplify oxidative stress and inflammatory cascade reactions through the accumulation of advanced glycation end products (AGEs) and their binding to the receptor RAGE (159), disrupt the normal metabolic reprogramming of macrophages, making them more inclined to maintain a pro-inflammatory state characterised by enhanced glycolysis, rather than differentiating into the M2 reparative phenotype that relies on oxidative phosphorylation and fatty acid oxidation. Persistent accumulation of ROS also disrupts the balance between the body’s oxidative and antioxidant systems, induces lipid peroxidation and reduces antioxidant enzyme activity, thereby on the one hand inhibiting osteoblast proliferation, differentiation and promoting their apoptosis, and on the other hand enhancing the bone resorption activity of osteoclasts, ultimately leading to bone loss and destruction of bone microstructure (160). Furthermore, impaired insulin signalling, increased bone marrow adiposity and mitochondrial dysfunction under diabetic conditions further deteriorate the bone marrow microenvironment, impair the osteogenic differentiation potential of bone marrow mesenchymal stem cells (BMSCs), and indirectly amplify macrophage-mediated chronic inflammatory responses (161). Diabetes also enhances the production of inflammatory cytokines such as TNF-α, IL-1β and IL-6 in macrophages, maintains a persistent chronic low-grade inflammatory state, and hinders their differentiation into the M2 reparative phenotype (162). Thus, the interactions between high glucose-induced metabolic abnormalities, oxidative stress and inflammatory amplification effects collectively drive macrophage functional imbalance, and participate in the onset and progression of DOP by promoting osteoclastogenesis and inhibiting osteogenesis.

## 4 Targeted macrophage regulation strategies for osteoporosis

Osteoporosis is one of the most common chronic skeletal diseases worldwide, which seriously threatens the quality of life and health status of middle-aged and elderly populations. Currently, its therapeutic strategies mainly include basic interventions (such as lifestyle modification, calcium and vitamin D supplementation), pharmacotherapy, rehabilitation exercise and others, but these methods still have certain limitations. In recent years, with the deepening of research on the regulatory mechanisms of osteoimmunology, macrophages, as a crucial hub for maintaining bone microenvironment homeostasis and regulating bone remodelling, their polarisation imbalance under pathological conditions has been recognised as one of the key mechanisms driving the onset and progression of osteoporosis. On this basis, intervention strategies targeting macrophages have gradually become a research hotspot. For instance, approaches such as inducing macrophage polarisation towards the M2 phenotype and achieving targeted delivery in combination with biomaterials have demonstrated the potential for multi-target synergistic regulation and precise therapy in preclinical studies (163, 164). Although most relevant studies are still in the stage of animal experiments and face challenges such as safety evaluation, clinical translation efficiency and standardised application, they still provide a new theoretical basis and intervention direction for breaking through the bottlenecks of traditional treatment and improving the prevention and treatment efficacy of osteoporosis.

### 4.1 Targeting macrophage polarisation

Targeting macrophage polarisation is an innovative direction for the treatment of osteoporosis, whose core revolves around the imbalance of macrophage M1/M2 polarisation. Under pathological conditions, macrophages tend to polarise towards the M1 phenotype, activate osteoclasts and inhibit osteoblasts by secreting factors such as TNF-α and IL-6 (165), exacerbating bone loss; however, targeted strategies restore the balance precisely by regulating the direction of macrophage polarisation. Studies have shown (166) that IL-4 is an important regulatory factor for correcting macrophage polarisation imbalance and improving bone metabolism. In macrophages, IL-4 binds to its receptor and promotes JAK1 phosphorylation, thereby inducing macrophage polarisation towards the M2 phenotype, while facilitating the nuclear translocation of downstream STAT6 and upregulating the expression of M2-specific target genes, further reinforcing the characteristics of the M2 phenotype (167). Furthermore, under IL-4 stimulation, the expression of osteogenesis-related factors such as IGF-1 and VEGF in M2 macrophages is significantly increased (168). Nevertheless, it should be noted that the current evidence in the field of targeting macrophage polarisation is still dominated by cellular and animal experiments, and studies that have truly entered the clinical translation stage remain limited. Meanwhile, a growing number of single-cell studies suggest (169) that although the traditional M1/M2 dichotomy helps explain the mechanism of macrophage polarisation, it is difficult to fully capture the continuous heterogeneity and dynamic plasticity exhibited by bone tissue macrophages during bone repair. These studies based on mononuclear transcriptome analysis have found that multiple macrophage subsets with continuous transition characteristics exist after bone injury, rather than the simple two discrete states of M1 or M2. Future research should combine single-cell sequencing, spatial transcriptomics and multi-omics analysis of the bone microenvironment to further clarify the functional division of macrophage subsets at different disease stages and different bone sites, as well as their interaction networks with osteoblasts, osteoclasts and BMSCs, thereby providing a more solid theoretical basis for the precision immunotherapy of osteoporosis.

### 4.2 Traditional pharmacotherapies for osteoporosis

For patients diagnosed with osteoporosis by DXA bone densitometry, those who have sustained fragility fractures at sites such as the vertebrae or hip, and those with osteopenia but high fracture risk, effective anti-osteoporosis pharmacotherapy can increase bone mineral density, improve bone quality, and significantly reduce the risk of fracture occurrence. Traditional anti-osteoporosis drugs can be classified into anti-resorptive agents, anabolic agents, dual-action agents, and drugs with other mechanisms according to their mechanisms of action (170) (see **Table 2**). Notably, in addition to directly acting on osteoclasts and osteoblasts, traditional anti-osteoporosis drugs can also affect macrophage polarisation by remodelling the osteoimmune microenvironment; however, the regulatory direction of different drugs on the M1/M2 balance is not consistent, and current evidence is still dominated by cellular and animal experiments. Among anti-resorptive agents, bisphosphonates (e.g., alendronic acid, risedronic acid, ibandronic acid, and zoledronic acid) not only inhibit osteoclasts but also suppress the mevalonate pathway in the monocyte-macrophage/osteoclast lineage, block protein prenylation, and alter small GTPase signalling (171), which constitutes an important molecular basis for their inhibition of bone resorption. Nevertheless, their effects on macrophage polarisation are not consistently anti-inflammatory. Studies have shown (172) that zoledronic acid promotes macrophage polarisation towards the M1 phenotype and inhibits the M2 phenotype via the TLR4/NF-κB signalling pathway, accompanied by increased pro-inflammatory cytokines; this persistent pro-inflammatory state is thought to be associated with the occurrence of adverse reactions such as bisphosphonate-related osteonecrosis of the jaw (BRONJ). Parathyroid hormone (PTH), as an anabolic agent, can promote the repair of jaw bone defects and fracture healing, and reduce inflammatory responses. Relevant studies have demonstrated (173) that PTH downregulates the expression of miR-155 in macrophages, leading to increased expression of its target gene SOCS1, which further reduces the proportion of M1 macrophages, thereby attenuating inflammation and enhancing bone regeneration. Denosumab is a RANKL inhibitor, a fully humanised monoclonal antibody specific for RANKL (174), which inhibits the binding of RANKL to its receptor RANK, reduces osteoclast formation, function, and survival, thereby decreasing bone resorption, increasing bone mineral density, improving the strength of cortical and trabecular bone, and reducing the risk of fractures.

**Table 2:** Classification of traditional anti-osteoporosis drugs.

Drug classification	Mechanism of action	Representative drugs
Bone resorption inhibitors	Inhibit the formation and activity of osteoclasts or induce their apoptosis, reduce the rate of bone resorption, and maintain bone mass stability	1.Bisphosphonates: Alendronate Sodium, Zoledronic Acid, etc. 2.RANKL Inhibitors: Denosumab 3.Selective Estrogen Receptor Modulators (SERMs): Raloxifene 4.Calcitonins: Salmon Calcitonin, Elcatonin, etc.
Bone formation promoters	Activate the activity of osteoblasts, promote the synthesis and mineralization of bone matrix, and increase bone mass and bone strength	1.Parathyroid Hormone (PTH) Analogues: Teriparatide 2.Sclerostin Inhibitors: Romosozumab
Dual-action drugs	Simultaneously inhibit bone resorption and slightly promote bone formation, thereby bidirectionally regulating the balance of bone metabolism	Strontium Salts: Strontium Ranelate
Drugs with other mechanisms	Exert effects through mechanisms that do not directly inhibit bone resorption or promote bone formation, such as regulating calcium-phosphorus metabolism and improving the bone microenvironment	1.Active Vitamin D and Its Analogues: Calcitriol, Alfacalcidol, etc. 2.Calcium Supplements
Chinese patent medicines	Tonifying the Liver and Kidney, Strengthening the Spleen and Benefiting Qi, Promoting Blood Circulation to Remove Blood Stasis	1.Drynaria Rhizome Total Flavonoid Preparations 2.Epimedium Herb Total Flavonoid Preparations 3.Artificial Tiger Bone Powder Preparations 4.Traditional Chinese Medicine Compound Preparations

### 4.3 Emerging intervention technologies

Mesenchymal stem cells (MSCs) are multipotent cells involved in tissue repair and immunomodulation, which can regulate immune responses and exert anti-inflammatory effects in specific microenvironments through multiple pathways (175). MSCs can modulate macrophage polarisation via mechanisms including paracrine secretion of soluble factors, exosome release, metabolic reprogramming and mitochondrial transfer, inhibiting the formation of M1 macrophages and promoting polarisation towards the M2 phenotype, thereby alleviating local inflammatory responses and promoting tissue repair (176, 177). In recent years, the crosstalk between MSCs and macrophages has received increasing attention in disease progression and the maintenance of inflammatory microenvironment homeostasis. Studies have found that MSCs regulate cytokine secretion by macrophages in an inflammatory environment, reducing pro-inflammatory cytokines and immune-related cytokines while increasing the levels of anti-inflammatory cytokines (IL-10) and growth factors (VEGF), and inhibit M1 macrophage polarisation by modulating the NF-κB signalling pathway, thus alleviating bone loss (178). In recent years, although biomaterials have been widely used in clinical bone repair, they cannot participate in the regulation of the osteoimmune microenvironment. Therefore, designing biomaterials that specifically target and regulate macrophage function provides a promising therapeutic strategy for improving osteoporotic bone repair. Wang X et al. developed a PMVGX composite material that can serve as a scaffold for cell adhesion, vascular ingrowth and bone tissue regeneration; this biomaterial inhibits bone marrow inflammation by promoting M2 macrophage polarisation, while also enhancing macrophage activation and clearing apoptotic cells, creating favourable conditions for the restoration of the microenvironment (179). The combined use of three-dimensional (3D) printed scaffolds and bioactive factors is an emerging strategy for the treatment of bone defects. Animal experiments have shown (180) that 3D cryoprinted scaffolds exert therapeutic effects of osteogenesis-angiogenesis coupling and macrophage phenotype regulation, which can promote bone defect regeneration. Thus, the integration of nanotechnology, biomaterials and cell biology has emerged as a promising frontier for advancing bone regeneration therapies, aiming to regulate the balance of macrophage polarisation and thereby create a microenvironment conducive to bone regeneration.

## 5 Research challenges and future prospects

One significant risk of targeting macrophages for the treatment of osteoporosis is the potential induction of immune regulatory imbalance. Macrophages play a crucial regulatory role in the immune system, and alterations in their polarisation status can disrupt the balance of the entire immune response. Excessive inhibition of M1 macrophage polarisation during treatment, while reducing inflammatory damage to bone tissue, may simultaneously impair the body’s immune defence capacity, making it more susceptible to pathogenic infections. In some studies, the use of specific inhibitors to block the NF-κB signaling pathway and inhibit M1 macrophage polarisation, although alleviating inflammation-related bone damage to a certain extent, significantly increased the susceptibility of experimental animals to bacterial infections. While M2 macrophages promote tissue repair, they may also lead to enhanced immune tolerance, reducing the body’s immune surveillance capacity against abnormal cells such as tumour cells. In the tumour microenvironment, tumour-associated macrophages (TAMs) often exhibit an M2 polarisation phenotype, and they help tumour cells evade immune system attack by secreting immunosuppressive factors and other mechanisms (181). Meanwhile, immune regulatory imbalance may also trigger autoimmune diseases, such as rheumatoid arthritis and systemic lupus erythematosus.

Although targeting macrophages for the treatment of osteoporosis holds significant promise, its further development still faces key challenges posed by disease and patient heterogeneity. First, macrophage polarisation is not a static M1/M2 dichotomy but is co-regulated by multiple factors including osteoporosis aetiology, disease stage and age-related immunosenescence, exhibiting continuous and dynamic characteristics. This suggests that future precision therapeutic strategies may require patient stratification based on the characteristics of the bone marrow immune microenvironment to identify populations more likely to benefit from specific immunomodulatory interventions. Second, osteoporosis itself exhibits marked heterogeneity, and different patients may have distinct molecular profiles or immunological subtypes, such as heterogeneous S subtypes, which may lead to differences in the activation status of macrophage-related pathways and treatment responses, thereby increasing the complexity of clinical translation. Furthermore, most current evidence on macrophage-targeted therapies is still mainly derived from animal models and *in vitro* experiments, and these preclinical studies cannot fully simulate the complex etiological composition, comorbidity backgrounds and long-term chronic inflammatory states of human osteoporosis, so there are still limitations in extrapolating their results to human diseases. Future research should further integrate single-cell sequencing, spatial transcriptomics and multi-omics analytical approaches to elucidate the functional status and spatiotemporal distribution characteristics of macrophages in the bone microenvironment of different patient subgroups, and combine clinical cohorts to validate stratification biomarkers and therapeutic targets, so as to advance macrophage-targeted strategies towards more precise and translatable intervention paradigms for osteoporosis.

How to achieve precise spatiotemporal regulation during bone remodeling may represent another key challenge. Since macrophage phenotypes are dynamically influenced by local inflammatory signals and changes in the bone microenvironment, and exert distinct functions at different stages of bone resorption and bone formation, static or sustained polarisation regulation strategies may fail to effectively restore bone homeostasis and may even disrupt the normal bone remodeling process. On this basis, future research urgently needs to develop intelligent regulatory systems responsive to changes in the bone microenvironment to achieve on-demand and precise modulation of macrophage polarisation status at different stages of bone remodeling. For example, constructing delivery platforms with temporally controlled release and microenvironment-responsive capabilities by integrating local features such as inflammatory cytokine levels, pH changes, ROS accumulation, enzymatic signals or mechanical stimuli may selectively inhibit pro-inflammatory phenotypes during the active phase of bone resorption, while promoting regeneration-favourable immunomodulatory phenotypes during the phase of bone formation and tissue repair, thereby restoring bone homeostasis more effectively.

Another key challenge is the difficulty in delivering drugs precisely to the target site. Macrophages are widely distributed in all tissues and organs throughout the body, making it extremely challenging to make drugs specifically act on macrophages in bone tissue without affecting macrophages in other sites and normal tissues. Currently commonly used drug delivery systems, such as nanoparticles and liposomes, although they can improve the targeting efficiency of drugs to a certain extent (182), still struggle to achieve precise localisation of macrophages in bone tissue. The *in vivo* distribution of nanoparticles is affected by multiple factors, including particle size, surface charge and blood circulation time, which make it difficult for nanoparticles to accurately accumulate around macrophages in bone tissue, resulting in a low drug utilisation rate. Constructing macrophage-targeted drug delivery systems based on nanotechnology has become one of the current research hotspots (183). Through surface modification of nanoparticles, they can specifically recognise and bind to receptors on the macrophage surface, thereby achieving precise drug delivery. Mannose-modified nanoparticles can target and bind to mannose receptors on the macrophage surface, efficiently delivering encapsulated drugs into macrophages. Nanotechnology can also realise the co-delivery of multiple drugs; by rationally designing the structure and composition of nanoparticles, drugs with different mechanisms of action can be simultaneously encapsulated within nanoparticles to achieve multi-target synergistic therapy for macrophages. Research on macrophages in osteoporosis involves multiple disciplinary fields, and multidisciplinary collaboration among medicine, biology, materials science and other disciplines is crucial for advancing research progress in this field. Through the cross-integration of multiple disciplines, the strengths of each discipline can be fully leveraged to jointly conduct research on macrophages in osteoporosis and promote the innovation and development of diagnostic and therapeutic technologies.

## 6 Conclusion

The pathological mechanisms of osteoporosis have been further expanded from the traditional bone cell functional imbalance to osteoimmune microenvironment dysregulation. As key regulatory cells in the osteoimmune microenvironment, the M1/M2 polarisation balance of macrophages is of great significance for maintaining bone metabolic homeostasis. Under physiological conditions, macrophages form a complex and tight interactive network with osteoblasts, osteoclasts and bone marrow mesenchymal stem cells (BMSCs) through the dynamic switching of polarisation phenotypes, thereby ensuring the dynamic balance between bone resorption and bone formation. However, under the pathological conditions of the onset and progression of osteoporosis, factors such as hormone deficiency, ageing and metabolic abnormalities can lead to macrophage polarisation imbalance, manifested as excessive accumulation of M1 macrophages and impaired function of M2 macrophages, which in turn promotes the sustained progression of bone metabolic disorders by enhancing osteoclast activation, inhibiting the osteogenic function of osteoblasts and exacerbating local inflammatory responses. Based on the in-depth understanding of the mechanism of macrophage polarisation imbalance, this paper systematically summarizes macrophage-targeted intervention strategies, aiming to provide new research ideas, potential therapeutic targets and theoretical basis for the prevention and treatment of osteoporosis, and lay a foundation for subsequent clinical translation research.
